# Factors associated with knowledge of mother-to-child transmission of HIV among reproductive-age women in Indonesia: a multilevel analysis

**DOI:** 10.1186/s12981-024-00596-6

**Published:** 2024-02-20

**Authors:** Agani Afaya, Aloysia Ispriantari

**Affiliations:** 1 College of Nursing, Yonsei University, Seoul, South Korea; 2 Department of Nursing, School of Nursing and Midwifery, University of Health and Allied Sciences, Ho, Ghana; 3 Department of Nursing, Institute Technology, Science and Health RS dr Soepraoen, Malang, Indonesia

**Keywords:** MTCT, HIV, IDHS, Indonesia

## Abstract

**Background:**

Maternal transmission of human immunodeficiency virus (HIV) commonly occurs from mother to child during pregnancy, delivery, and breastfeeding which accounts for almost all the new HIV infections among children aged 0–14 years. Despite major efforts and progress in controlling and preventing HIV, it continues to pose a great public health threat, especially in Indonesia. This study assessed the factors associated with the knowledge of mother-to-child transmission (MTCT) of HIV among reproductive-age women in Indonesia.

**Methods:**

This study used data from the 2017 Indonesian Demographic and Health Survey **(**IDHS). We sampled 39,735 reproductive-age women (15–49 years) for analysis. Using Stata version 16.0, multilevel logistic regression models were fitted, and the results were presented as adjusted odds ratios (aORs) with their confidence intervals (CIs).

**Results:**

The study found that 72% of women had knowledge of MTCT of HIV. Women who were aged between 45 and 49 years (aOR = 1.65, 95%CI = 1.46–1.88) had higher odds of knowledge of MTCT of HIV than those aged 15–19 years. Women who attained higher education (aOR = 2.92, 95%CI = 2.06–4.15) had increased odds of knowledge of MTCT of HIV than those with no formal education. Women who had four children (aOR = 1.19, 95% CI = 1.05–1.35) had higher odds of knowledge of MTCT of HIV than nulliparous women. Women who frequently read newspapers/magazines (aOR = 1.14, 95%CI = 1.06–1.25) and frequently used the internet almost every day (aOR = 1.28, 95%CI = 1.19–1.38) had higher odds of knowledge of MTCT of HIV than those who did not read newspapers/magazines and non-users of internet, respectively. Women within the richer (aOR = 1.11, 95%CI = 1.02–1.20) and the richest (aOR = 1.14, 95%CI = 1.04–1.25) wealth quintile higher odds of knowledge of MTCT of HIV than those in the poorest wealth quantile. Women who resided in rural areas were less likely to have knowledge of MTCT of HIV (aOR = 0.79, 95% CI = 0.74–0.86) than those in urban settlements.

**Conclusions:**

Knowledge of MTCT of HIV was slightly above average. The study findings on the factors associated with knowledge of MTCT of HIV provide evidence for policymakers and clinicians to utilize in the quest to eliminate MTCT of HIV among children. We recommend that awareness programs should consider the key findings from this study when delivering public education or when developing interventions to improve women’s knowledge on MTCT of HIV.

## Introduction

Globally, the reduction of new human immunodeficiency virus (HIV) infections have seen slow progress, particularly a decline of 23% from 2010 to 2019 [1]. Approximately, an average of 1.7 million new HIV infections were reported worldwide ranging from 1.2 million to 2.2 million [1]. The global quest to eliminate new HIV infections among children (aged 0–14 years) has stalled leading to an unacceptably high number of adolescent girls and young women (aged 10–24 years) acquiring HIV each year. In 2020, children accounted for 5% of all persons living with HIV but comprised of 15% of all people who died from AIDS-related causes [2]. Despite major efforts and progress in controlling and preventing HIV, it continues to pose a great public health threat, especially in Southeast Asia [3]. In southeast Asia, it is estimated that every year about 1400 babies are infected with HIV through mother-to-child transmission (MTCT) [4].

HIV pandemic patterns in Indonesia are intricate. In Indonesia, the HIV pandemic is mainly concentrated in key populations in most areas. However, there is a low-level widespread epidemic only in Papua province [5]. In 2019, Indonesia was observed to have the fastest-growing HIV epidemic among the adult population above 15 years [3, 5]. The estimated proportion of people living with HIV in Indonesia was 543,100 in 2020 while the proportion of women with HIV was 33% and 37% with AIDS. The prevalence of HIV among children under the age of four was 1.5%, and AIDS was 1% [6]. In areas with high HIV prevalence, all pregnant women are tested for HIV and syphilis during antenatal care until delivery. In areas with low HIV prevalence, testing is prioritized for pregnant women with sexually transmitted infections (STIs) or those at risk of HIV, STIs, and tuberculosis [7]. In 2021, 15% (1,520) of pregnant women with HIV received antiretroviral drugs; the final transmission rate, including breastfeeding period was 30.8%; and infants born to HIV positive mother had a virological test within two months of delivery (5.2%) [8]. This evidence shows that HIV transmission from mother to child still exists.

Maternal transmission of HIV commonly occurs from mother to child during pregnancy, delivery, and breastfeeding which accounts for almost all the new HIV infections among children aged 0-14years [9, 10]. It was indicated that without the appropriate interventions to prevent MTCT, the risk of transmission would range from 15 to 45%; meaning 5–10% during pregnancy, 10–20% during delivery, and 10–20% through mixed infant feeding [11]. However, the implementation of successful HIV/AIDS programs will decrease the rate to less than 5% [11].

Although Indonesia implemented the Prevention of Mother-to-Child Transmission (PMTCT) program, MTCT of HIV remains a challenge. Therefore, concerted efforts are required to raise awareness of reproductive-age women on PMTCT. Significant evidence shows that knowledge of MTCT of HIV has a positive impact on the PMTCT [12, 13]. Inadequate knowledge of PMTCT could make women more vulnerable to HIV/AIDS [14]. The population of women who are not key populations is estimated to make 35% contributions, the most significant, to the new HIV infections in 2024 [15]. It is therefore prudent in raising the awareness of reproductive-age women on MTCT of HIV. Therefore, identifying factors associated with reproductive women’s knowledge of MTCT of HIV would be key in providing public health education and strategies to combat MTCT of HIV.

A plethora of factors have been identified to be associated with women’s knowledge of MTCT of HIV which include the place of residence, occupation, socioeconomic status, exposure to mass media, and maternal education [16]. However, there are limited studies in Indonesia that use nationally representative data to assess reproductive age women’s knowledge of MTCT of HIV. Therefore this study assessed the factors associated with knowledge of MTCT of HIV among women of reproductive age in Indonesia. The findings from this study could contribute to existing programs and policy development to enhance women’s knowledge of MTCT of HIV which will further increase the prevention of MTCT of HIV in Indonesia.

## Methods

### Data source

This study used nationally representative data from the 2017 Indonesian Demographic and Health Survey (IDHS). Indonesia is a country with the fourth largest population in the world, with around 271 million people. The 2017 IDHS is the eighth survey in Indonesia conducted under the auspices of the Demographic and Health Surveys (DHS) Program. The government of Indonesia financed the 2017 IDHS with technical support from the ICF, which was funded by the U.S. Agency for International Development (USAID), which provides financial and technical support for developing countries to undertake DHS. The survey covered all 34 provinces in Indonesia and collected data on the maternal and child health indicators including knowledge of HIV, contraceptive use, fertility, antenatal and postnatal care, child health and nutrition, childhood immunization, marriage and sexual activity, fertility preferences, and other health issues.

The 2017 IDHS sample was selected using a multistage (two-stage stratified) sampling design. The first stage involved the selection of census blocks with a systematic proportional to size approach in line with the 2010 population census household list. The second stage involved the selection of households from each selected census block with systematic random sampling. In each household, women of reproductive age (15-49years) and men aged 15–54 were eligible for individual interviews. The survey used 49,261 households of which interviews were completed in 97% of households in urban areas and 98% of households in rural areas with a 1% non-response rate each. In the current study, 39,735 weighted samples of reproductive-age women (15-49years) were selected for analysis.

### Measures

#### Outcome measure

The outcome measure for this study was reproductive women’s knowledge of MTCT of HIV. In the IDHS, three key questions were captured to assess the knowledge of reproductive-age women on MTCT of HIV. The questions were as follow: (a) “Can HIV virus be transmitted from a mother to her baby during pregnancy?” (b) “Can HIV virus be transmitted from a mother to her baby during delivery?” (c) “Can HIV virus be transmitted from a mother to her baby by breastfeeding?”. Women who responded affirmatively to all the three questions were considered to have knowledge of MTCT of HIV and were coded as 1, otherwise, they were considered as not having knowledge of MTCT and were coded as 0. Respondents who indicated “don’t know” were treated as a lack of knowledge.

### Explanatory variables

The explanatory variables were selected based on the review of related literature on factors associated with women’s knowledge of MTCT of HIV [1, 17, 18] and were categorized as individual and contextual level factors. The individual level factors included age, marital status, educational level, occupation, health insurance coverage, parity, frequency of listening to radio, frequency of reading newspaper or magazine, frequency of watching television, and frequency of using internet last month. The contextual level factors included distance to health facility, sex of household head, household wealth index and type of residence. The individual-level factors included age of the respondent which was categorized as 15–19, 20–24, 25–29, 30–34, 35–39, 40–44, and 45–49 while marital status was re-categorized as not married and married. Education level was categorized as no formal education, primary school, secondary school, and higher. The rest of the other variables were categorized as follows: occupation (working and not working); health insurance coverage (no and yes); parity (0, 1, 2, 3, and ≥ 4); frequency of listening to radio (“not at all”, “less than once a week” and “at least once a week”); frequency of reading newspaper or magazine (“not at all”, “less than once a week” and “at least once a week”); and frequency of watching television (“not at all”, “less than once a week” and “at least once a week”); frequency of using internet last month (“not at all”, “less than once a week”, “at least once a week” and “almost every day”). The contextual level factors such as distance to health facility was categorized as “not a big problem” and “a big problem”; sex of household head was categorized as male and female; household wealth index was grouped as poorest, poorer, middle, richer, and richest and the type of residence was coded as urban and rural.

### Statistical analysis

Statistical analysis was conducted using Stata software version 16.0. Descriptive statistics such as frequencies and percentages were used to describe the sample and present the prevalence of knowledge of MTCT of HIV among reproductive women. Chi-square test was conducted to show the distribution and associations between the explanatory and outcome variable. A multicollinearity diagnostic test was conducted for the explanatory variables and none of the variables had a Variance Inflation Factor (VIF) of more than 10. Based on the rule of thumb, none of the variables had a VIF suggestive of harmful collinearity, so therefore none was eliminated from further analysis [19]. The statistically significant explanatory variables were moved into the multilevel binary logistic regression analysis. Four models were fitted to present the results. Model 0 was fitted without explanatory variables, also known as the random intercept (empty model). The second model (model I) was fitted using the individual level variables while the third model (model II) comprised of the contextual level factors. The final model (model III) was the combination of all the explanatory variables against women’s knowledge of MTCT of HIV. The multilevel logistic regression analysis consisted of random effects and fixed effects [20]. The fixed effects results of the models were presented as Adjusted Odds Ratios (aORs) with confidence intervals (CI). The intra-class correlation (ICC) was used to assess the random effects. The log-likelihood and Akaike information criterion (AIC) test were used for the model comparison. The sample data for the study were weighted (v005/1000000) and to account for the complex sampling nature of the survey, the command ‘*svy’* in Stata was used in the regression analyses.

### Ethical consideration

Ethical approval for the survey was granted by the Institutional Review Board of ICF International. MEASURE DHS Program granted access for us to use the dataset for this study. The dataset is available at https://dhsprogram.com/data/available-datasets.cfm upon request. The survey sought informed consent from all the participants during data collection. The current study adheres to all the ethical standards and guidelines for conducting human research and in line with the Declaration of Helsinki ethical principles.

## Results

### Sociodemographic characteristics of respondents

Table 1 depicts the distribution of respondents’ characteristics. This study included a weighted sample of 39,735 reproductive-age women for analysis. The majority of the women (15.6%) were between the ages of 35-39 years while 70.0% were married. Most of the women (61.2%) attained secondary education and 58.7% of them were employed. Approximately 59.2% of women who were of reproductive age had health insurance, and the majority of them (90.4%) said that getting to the medical facility wasn’t a major issue. Approximately 25.0% of the women were from the richest household and about 56.3% of the women resided in urban settings (Table 1).

### Knowledge of MTCT of HIV among reproductive age women

**Fig. 1:**
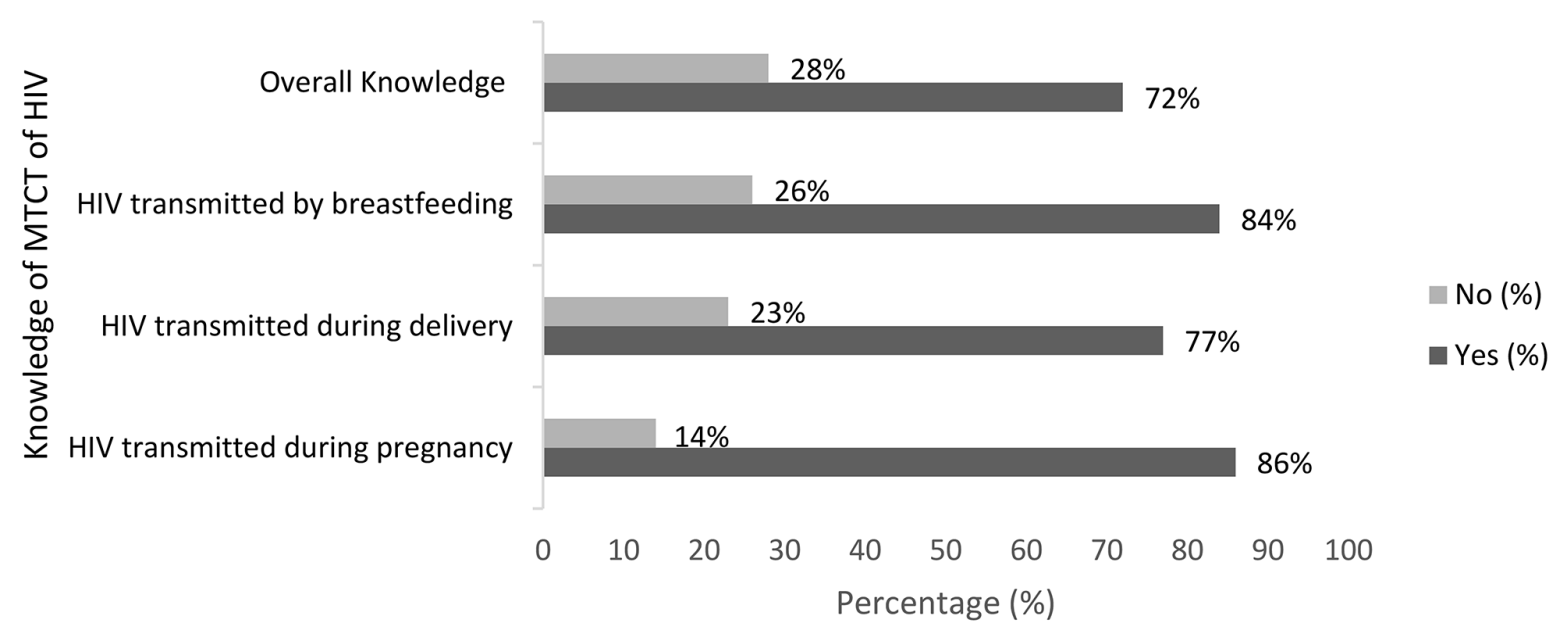
Knowledge of MTCT of HIV among women in Indonesia

Figure 1 shows the level of knowledge of MTCT of HIV among reproductive-age women in Indonesia. The overall knowledge of MTCT of HIV was 72% (70.9–72.3). About 86% of the women knew that HIV is transmitted during pregnancy while 77% knew that HIV is transmitted during pregnancy. Most women (84%) also knew that HIV is transmitted by breastfeeding.

The knowledge of MTCT of HIV was higher among the age group of 35–39 compared to the ages between 15-19years. Women who were marred had the highest proportion of knowledge of MTCT of HIV compared to the women not married. Knowledge of MTCT of HIV was higher among those with higher education compared with women with no formal education. Women who had health insurance had a slightly higher proportion of knowledge of MTCT of HIV compared with those without health insurance. Women within the richest wealth quintile had the highest proportion of knowledge of MTCT of HIV compared with those in the poorest wealth quintile. Women who resided in urban areas had a higher proportion of knowledge of MTCT of HIV compared with their rural counterparts (Table 1).

**Table 1:** Distribution of sociodemographic characteristics of respondents and MTCT of HIV

Variables	Weighted N (39,735)	Weighted %	MTCT of HIV		
			No (%)	Yes (%)	***p***-value
**Age (years)**					< 0.001
15–19	6,427	16.2	36.0	64.0	
20–24	5,874	14.8	28.5	71.5	
25–29	5,621	14.1	28.4	71.6	
30–34	5,895	14.9	27.0	73.0	
35–39	6,216	15.6	25.0	75.0	
40–44	5,253	13.2	26.0	74.0	
45–49	4,449	11.2	26.4	73.6	
**Marital status**					< 0.001
not married	11,914	30.0	31.3	68.7	
Married	27,821	70.0	27.1	72.9	
**Educational status**					< 0.001
No education	161	0.4	44.5	55.5	
Primary	7,799	19.6	35.5	64.5	
Secondary	24,308	61.2	28.0	72.0	
Higher	7,467	18.8	21.7	78.3	
**Occupation**					0.062
Not working	16,398	41.3	29.0	71.0	
Working	23,337	58.7	27.8	72.2	
**Health insurance**					
No	16,202	40.8	29.8	70.2	
Yes	23,533	59.2	27.4	72.6	
**Parity**					< 0.001
**0**	12,530	31.5	31.6	68.4	
1	8,019	20.2	28.1	71.9	
2	10,478	26.4	25.4	74.6	
**3**	5,488	13.8	26.3	73.7	
≥ 4	3,220	8.1	29.7	70.3	
**Frequency of listening to radio**					0.022
Not at all	22,213	55.9	28.9	71.1	
Less than once a week	11,641	29.3	27.8	72.2	
At least once a week	5,881	14.8	27.6	72.4	
**Frequency of reading newspaper or magazine**					< 0.001
Not at all	21,547	54.2	29.8	70.2	
Less than once a week	13,529	34.1	27.7	72.3	
At least once a week	4,659	11.7	23.7	76.3	
**Frequency of watching television**					0.693
Not at all	860	2.2	31.9	68.1	
Less than once a week	4,779	12.0	29.1	70.9	
At least once a week	34,096	85.8	28.2	71.8	
**Frequency of using internet last month**					< 0.001
Not at all	17,657	44.4	31.0	68.9	
Less than once a week	1,111	2.8	32.6	67.4	
At least once a week	3,568	9.0	30.4	69.6	
Almost every day	17,399	43.8	24.9	75.1	
**Distance to health facility**					0.038
Big problem	3,821	9.6	30.5	69.5	
Not a big problem	35,914	90.4	28.1	71.9	
**Sex of household head**					0.810
Male	34,984	88.0	28.6	71.4	
Female	4,751	12.0	26.8	73.2	
**Wealth index**					< 0.001
Poorest	5,293	13.3	31.7	68.3	
poorer	7,199	18.1	31.4	68.6	
Middle	8,178	20.6	29.5	70.5	
Richer	9,126	23.0	27.3	72.7	
Richest	9,939	25.0	24.4	75.6	
**Type of place of residence**					< 0.001
Urban	22,374	56.3	25.4	74.6	
Rural	17,361	43.7	32.1	67.9	

### Factors associated with knowledge of MTCT HIV among reproductive age women

#### Fixed effects (measures of associations) results

Table 2 depicts results from the multilevel analysis of the factors associated with MTCT of HIV among women in Indonesia after adjusting for other covariates. In the final model, we found that increasing age was associated with higher knowledge of MTCT of HIV. Specifically, women between the ages of 45–49 years had higher odds of knowledge of MTCT of HIV than those aged 15–19 years (aOR = 1.65, 95%CI = 1.46–1.88). We observed from the analysis that increasing educational level was associated with higher knowledge of MTCT of HIV. In particular, women with higher educational levels were over five times more likely to have knowledge of MTCT of HIV (aOR = 2.92 95%CI = 2.06–4.15) than those with no education. Women who had children were more likely to have knowledge of MTCT of HIV. Women who had four children had higher odds of knowledge of MTCT of HIV (aOR = 1.19 95%CI = 1.05–1.35) than nulliparous women. Women who frequently read newspapers/magazines (aOR = 1.14, 95%CI = 1.06–1.25) at least once a week were more likely to have knowledge of MTCT of HIV compared with those who never read newspapers/magazines. Women who frequently used the internet almost every day (aOR = 1.28, 95%CI = 1.19–1.38) were more likely to have knowledge of MTCT of HIV than non-users. An increasing level of wealth index was associated with higher knowledge of MTCT of HIV. Women within the richer (aOR = 1.11, 95%CI = 1.02–1.20) and the richest (aOR = 1.14, 95%CI= [1.04–1.25) wealth quintile were more likely to have knowledge of MTCT of HIV than those in the poorest wealth quantile. Women who resided in the rural areas were less likely to have knowledge of MTCT of HIV (aOR = 0.79, 95% CI = 0.74–0.86) than those in the urban settlement.

#### Random effects (measures of variations) results

From Table 2, the empty model showed a substantial variation in the likelihood of women’s knowledge about MTCT of HIV in Indonesia across the PSUs clustering (σ2 = 0.43 95%CI [0.38–0.48]). The empty model (Model 0) indicated that 11.6% of the variation in women’s knowledge of MTCT in Indonesia was attributed to the variation between-cluster characteristics, i.e., (ICC = 0.116). The variation between clusters decreased slightly to 11.4% in Model I, representing only the individual level model (Model I). In the contextual level model (Model II), the ICC decreased to 10.8%. There was a further slight decline (10.1%) of the ICC in the final Model (Model III). This further emphasize that the variations in the likelihood of women’s knowledge about MTCT in Indonesia are attributed to the clustering differences within PSUs. The AIC value showed a successive reduction, which means a substantial improvement in each of the models over the previous model and also affirmed the goodness of Model III developed in the analysis. Also, the best fit model was determined by the highest loglikelihood (-23110.426) value among the models. Therefore, the complete model (Model III) consisting of all the explanatory variables was selected to predict women’s knowledge of MTCT of HIV in Indonesia.

**Table 2:** Factors associated with knowledge of mother-to-child transmission of HIV among reproductive-age women

Variables	Model 0	Model I aOR [95% CI]	Model II aOR [95% CI]	Model III aOR [95% CI]
**Individual level factors**				
**Age (years)**				
15–19		1		1
20–24		1.27^***^[1.17,1.39]		1.27^***^[1.17,1.39]
25–29		1.28^***^[1.16,1.43]		1.27^***^[1.16,1.42]
30–34		1.44^***^[1.29,1.61]		1.41^***^[1.27,1.58]
35–39		1.61^***^[1.44,1.81]		1.56^***^[1.39,1.75]
40–44		1.67^***^[1.49,1.89]		1.61^***^[1.43,1.81]
45–49		1.74^***^[1.54,1.97]		1.65^***^[1.46,1.88]
**Marital status**				
not married		1		1
Married		1.03[0.95,1.13]		1.03[0.95,1.13]
**Educational status**				
No education		1		1
Primary		1.58^**^[1.12,2.23]		1.55^*^[1.10,2.20]
Secondary		2.42^***^[1.72,3.41]		2.33^***^[1.66,3.29]
Higher		3.07^***^[2.17,4.36]		2.92^***^[2.06,4.15]
**Health insurance**				
No		1		1
Yes		1.03[0.99,1.09]		1.03[0.99,1.09]
**Parity**				
**0**		1		1
1		1.18^***^[1.08,1.30]		1.19^***^[1.08,1.30]
2		1.29^***^[1.17,1.44]		1.29^***^[1.16,1.43]
**3**		1.28^***^[1.14,1.44]		1.27^***^[1.14,1.43]
≥ 4		1.18^**^[1.05,1.34]		1.19^**^[1.05,1.35]
**Frequency of listening to radio**				
Not at all		1[1.00,1.00]		1
Less than once a week		0.996[0.94,1.06]		0.99[0.94,1.05]
At least once a week		0.939[0.87,1.01]		0.932[0.87,1.00]
**Frequency of reading newspaper or magazine**				
Not at all		1		1
Less than once a week		1.04[0.99,1.11]		1.04[0.99,1.10]
At least once a week		1.16^***^[1.07,1.26]		**1.14** ^**^ **[1.06,1.25]**
**Frequency of using internet last month**				
Not at all		1		1
Less than once a week		1.10[0.97,1.25]		1.09[0.96,1.24]
At least once a week		1.20^***^[1.10,1.31]		1.16^***^[1.07,1.27]
Almost every day		1.36^***^[1.28,1.46]		1.28^***^[1.19,1.38]
**Contextual level factors**				
**Distance to health facility**				
Big problem			1	1
Not a big problem			0.99[0.92,1.08]	0.93[0.87,1.02]
**Household wealth index**				
Poorest			1	1
poorer			1.10^**^[1.03,1.20]	1.02[0.94,1.10]
Middle			1.242^***^[1.15,1.34]	1.076[0.99,1.17]
Richer			1.36^***^[1.26,1.48]	1.11^*^[1.02,1.20]
Richest			1.575^***^[1.45,1.71]	1.14^**^[1.04,1.25]
**Type of place of residence**				
Urban			1	1
Rural			0.69^***^[0.65,0.75]	0.79^***^[0.74,0.86]
**Random effect model**				
PSU (95%CI)	0.43[0.38–0.48]	0.42[0.37–0.47]	0.39[0.35–0.44]	0.41[0.36-0.46]
ICC	0.1167678	0.1140848	0.108298	0.1017918
Wald chi-square	Reference	754.05***	234.14***	792.84***
**Model fitness**				
Log-likelihood	-23521.453	-23131.761	-23404.466	-23110.426
AIC	47046.91	46311.52	46824.93	46280.85
BIC	47064.09	46517.68	46893.65	46538.55
N	39,735	39,735	39,735	39,735

Exponentiated coefficients; 95% confidence intervals in brackets; aOR = adjusted odds ratios; CI Confidence Interval; ^*^p < 0.05, ^**^p < 0.01, ^***^p < 0.001; 1 = Reference category; ICC = Intra-Class Correlation; AIC = Akaike’s Information Criterion, BIC = Bayesian information criterion

## Discussion

This study assessed the factors associated with the knowledge of MTCT of HIV among women of reproductive age in Indonesia. The study found that 72% of women in Indonesia had knowledge of MTCT of HIV. Our study finding is higher than several other studies conducted in Asia and sub-Saharan Africa (SSA). Within the Asian continent, a study conducted in Vietnam reported a knowledge level of 46.83%, which is lower than the present study [18]. Also, within SSA, certain studies from Zimbabwe [21], Tanzania [22], and Ethiopia [17] reported 70.5%, 46%, and 41.1%, respectively. Furthermore, an analysis of 33 sub-Saharan African countries also revealed a knowledge level of 56.21% which is also far lower than the current findings [16]. The higher knowledge level among women in this study could be attributed to the integration of the Prevention of Mother-to-Child Transmission (PMTCT) of HIV program into antenatal care clinics as a part of the national HIV programs by the MOH of Indonesia. In 2017, approximately 664 healthcare facilities were providing PMTCT programs all over Indonesia [23]. The program aimed at creating awareness of MTCT of HIV among reproductive-age women in Indonesia which might have contributed to the knowledge level of women in this study.

The multilevel fixed effects results showed that maternal age, educational level, parity, frequency of reading newspapers or magazines, frequency of using the internet, wealth index, and type of place of residence was associated with knowledge of MTCT of HIV among women of reproductive age in Indonesia.

We found that women’s age was associated with knowledge of MTCT of HIV. In particular, increasing age was associated with higher knowledge of MTCT of HIV. This finding is in line with studies conducted in Zimbabwe [21], Tanzania [22], and Vietnam [18]. The plausible reason for this finding could be that the younger women might not have pregnancy or childbirth experience, so they would be less exposed to this information, usually given during antenatal care or neonatal visits [22]. In Indonesia, youth reproductive health programs that provide information and education on reproductive health, including HIV/AIDS and STI, are included in the Youth Care Health Program (PKPR) service. However, not all public health centers in Indonesia have implemented this service [15]. Our findings suggest that efforts to support the existing program and new strategies to create the awareness of younger women especially those below ages of 20 years on MTCT of HIV are imperative in order to reduce MTCT of HIV.

Evidence shows that higher education is a universal variable for increased knowledge of HIV transmission, and prenatal education [24–26]. This study found that the higher the woman’s education, the higher the knowledge of MTCT of HIV. This finding resonates with several other studies conducted in Ethiopia [17, 27, 28], Vietnam [18], Cameroon [29] and Tanzania [22]. Women with higher education has the ability to seek for information and being able to process health information better than women who are not educated [28]. Education of women has the propensity to increase the acquisition of knowledge about maternal health services and enables women to seek and utilize healthcare services [1, 30, 31]. Evidence also shows that educated women who utilize healthcare services such as ANC and health facility delivery are more likely to have PMTCT knowledge [32].

Our study found that multiparous women were more likely to have knowledge of MTCT of HIV compared to nulliparous women. This is in line with several other previous studies [1, 22, 32]. Multiparous women may have received more information on MTCT of HIV during prenatal visits, perhaps leading to improved knowledge.

Furthermore, this study revealed that women who were exposed to mass media were more likely to have knowledge of MTCT of HIV. Precisely women who read newspapers/magazines and the use of the internet in daily bases was associated with knowledge of MTCT of HIV. Our study finding collaborates with findings in Ethiopia [17]. Also our findings resonates with a multi-country study conducted in SSA [16]. In Indonesia, internet users are accelerating yearly; in 2017, internet users were 54.68%, increasing to 77.02%. In 2020, 76.48% of Indonesian women became internet users, and the main reason for internet use was access to social media and news/information [33]. Our findings suggest that mass media outlets are key in the quest to eliminate MTCT of HIV by 2030 if much attention is given to propagating most educative programs on media about MTCT and PMTCT.

Consistent with extent of literature women from higher socioeconomic status are more likely to have higher knowledge of MTCT of HIV [17, 18, 22, 28, 34]. The reason for this finding might be that woman in the lower socioeconomic status might have less access to healthcare services or health facilities due financial barriers and this might impede them from acquiring health information [35]. This will further affect women’s inability to access maternal health services such as antenatal care [36, 37]. Another plausible reason might be that women from higher socioeconomic households are exposed to several opportunities such as access education, health services, and social media to obtain health information on MTCT of HIV [17, 22, 28]. In addition, wealthier women believe health is an investment in the future [18]. Most Indonesian people still view public health services as providing low-quality services from health professionals, complex procedures, and long waiting times. Therefore, high-income Indonesians prefer to go to private health services that provide faster and simple procedure even though they have to spend more money in this facility [38]. For this reason, they will have longer time to discuss with health professionals and have more health information about any health issue they wish to enquire about.

Finally, we found that women from rural settings were less likely to have knowledge about MTCT of HIV. This finding resonates with a recent study conducted in Ethiopia where rural residents were less likely to know about MTCT of HIV [17]. Some other studies have reported that women from urban settings had better knowledge of MTCT of HIV [12, 27, 39]. The plausible reason could be that women from rural settlements might not have access to some key social amenities such healthcare facilities where they can seek health care and possibly receive education on MTCT of HIV. Also, exposure to mass media outlets might have been limited and this could have also limited their knowledge on MTCT of HIV. Although 94% of provinces in Indonesia have met the standards in each district having at least one public health center, the people still have not been able to access it properly [6]. One of the reasons is the geographical difference in Indonesia. As the largest archipelago in the world, many remote islands are difficult to reach [40, 41]. While in urban areas, private health services are also available so that it is easier to access health services, rural areas still rely on public health centers [40]. Geographical conditions also make many rural areas, especially in the eastern region of Indonesia, not to have adequate internet access [42].

### Strengths and limitations of the study

The strength of this study is the use of a nationally representative sample to assess women’s knowledge of MTCT of HIV in Indonesia. Using large sample means the study findings are generalizable to the entire reproductive age women in Indonesia. The findings from this study are anticipated to contribute to the designing of new policies and strategies to improve on existing programs on the PMTCT of HIV in order to control the disease in Indonesia. Notwithstanding the strengths, the study is fraught with some limitations. First, due to the cross-sectional nature of the DHS data, we could not draw casual inferences. Lastly, the survey dataset was limited to questions on MTCT did not have a component to assess their knowledge on the prevention of MTCT of HIV.

## Conclusion

Our study found that the knowledge of MTCT of HIV among women of reproductive age was slightly above average. We found that the knowledge level of MTCT was associated with maternal age, educational level, parity, frequency of reading newspapers or magazines, frequency of using the internet, wealth index, and type of place of residence. These findings provide evidence for policy makers and clinicians to utilize in the quest to eliminate MTCT of HIV among children. We recommend that awareness programs should consider the key findings from this study when delivering public education or when developing interventions to improve women’s knowledge of MTCT of HIV.

## Data Availability

The datasets used for this study is openly available and can be accessed via https://dhsprogram.com/data/.

## Abbreviations

AIDS: Acquired Immune Deficiency Syndrome
HIV: human immunodeficiency virus
MTCT: Mother to child transmission
PMTCT: Prevention of mother to child transmission
IDHS: Indonesian Demographic and Health Survey
DHS: Demographic and Health Survey
aOR: adjusted odds ratio
CI: Confidence Interval
ICC: Intra-Class Correlation
AIC: Akaike’s Information Criterion
BIC: Bayesian information criterion
LGAs: Local Government Areas
EAs: Enumeration areas
VIF: Variance Inflation Factor
MOH: Ministry of Health
UNFPA: United Nations Population Fund
USAID: U.S. Agency for International Development
